# Simulation Model for Dynamics of Dengue with Innate and Humoral Immune Responses

**DOI:** 10.1155/2018/8798057

**Published:** 2018-04-11

**Authors:** S. D. Perera, S. S. N. Perera

**Affiliations:** Research and Development Center for Mathematical Modeling, Department of Mathematics, University of Colombo, Colombo, Sri Lanka

## Abstract

Dengue virus is a mosquito borne* Flavivirus* and the most prevalent arbovirus in tropical and subtropical regions around the world. The incidence of dengue has increased drastically over the last few years at an alarming rate. The clinical manifestation of dengue ranges from asymptomatic infection to severe dengue. Even though the viral kinetics of dengue infection is lacking, innate immune response and humoral immune response are thought to play a major role in controlling the virus count. Here, we developed a computer simulation mathematical model including both innate and adaptive immune responses to study the within-host dynamics of dengue virus infection. A sensitivity analysis was carried out to identify key parameters that would contribute towards severe dengue. A detailed stability analysis was carried out to identify relevant range of parameters that contributes to different outcomes of the infection. This study provides a qualitative understanding of the biological factors that can explain the viral kinetics during a dengue infection.

## 1. Background

Dengue virus (DENV) has emerged as the most prevalent arthropod-borne disease in humans worldwide, with an estimated 390 million individuals infected per year, leading to approximately 500,000 hospitalizations and 25,000 deaths [1]. Dengue occurs mainly in tropical and subtropical regions around the world and is transmitted to humans through the bite of an infected mosquito,* Aedes aegypti*. The etiological agent of the dengue virus is a RNA virus that belongs to the family of Flaviviridae viruses, of which there are four distinct closely related serotypes (DENV-1, DENV-2, DENV-3, and DENV-4) [2, 3]. Infection by one serotype provides lifelong immunity against that particular serotype but only partial and temporary cross-immunity to the other serotypes [4].

These four serotypes cause a variety of clinical symptoms ranging from asymptomatic infection to dengue fever (DF) to its more severe manifestations, dengue hemorrhagic fever (DHF) and dengue shock syndrome (DSS). DF is a self-limiting illness with onset of fever along with symptoms such as headaches, muscle or joint pain, and rash [5–8]. Only a few would proceed to severe dengue, DHF, a condition characterized by plasma leakage, that may lead to circulatory shock resulting in DSS. Without proper monitoring and immediate treatment, DHF and DSS can be fatal. Currently there are no specific treatments or vaccines available against dengue. Therefore early diagnosis, bed rest, and maintaining patient's body fluid volume are critical for the care of severe dengue.

The immune response to dengue infection plays an important role in controlling the virus. The human immune system is made up of two parts: the innate immune response and adaptive immune response. The nonspecific innate immune response provides immediate protection against an invading pathogen and is the first line of defense. Innate immune response produces type I interferon (IFN) mainly IFN-*α*/*β* that has been shown to induce resistance to infection in neighboring uninfected cells and limit the spread of the virus [2, 9, 10]. In addition, IFN has the ability to activate natural killer (NK) cells during early stage of infection, which can destroy infected cells [9, 11, 12]. In dengue, IFN is generally detected 24–48 hours after infection and correlates with the virus titer peak [13]. Also early activation of NK cells has been seen in dengue patients [14, 15].

Interferon produced by infected epithelial cells is important for the activation of the adaptive immune response [10, 16]. The adaptive immune system takes longer to respond but provides long term immunity against an invading pathogen [5, 17, 18]. The adaptive immune response consists of antibody-secreting B-cells (humoral immune response) and cytotoxic T-cells (cell-mediated immune response). Both are responsible in clearing the infection and providing lifelong immunity against a pathogen [3, 17–19].

**Figure 1:**
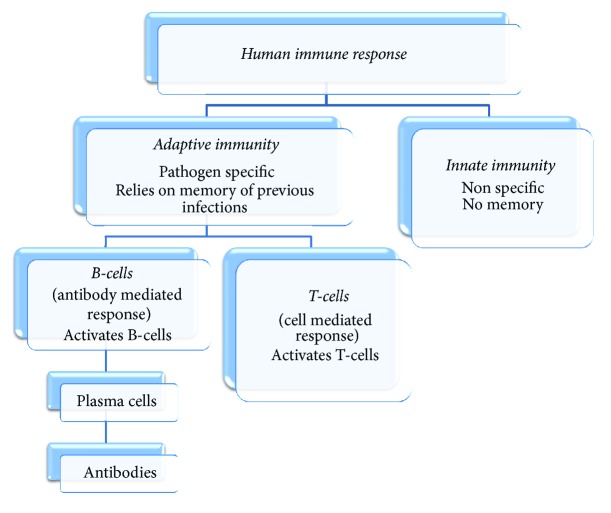
Human immune system.

As virions enter the body, they infect dendritic cells, macrophages, monocytes, and hepatocytes. As soon as the body learns that the cells are infected with dengue virus, it triggers the innate immune response. When the innate immunity is unable to curb the infection, it initiates the adaptive immune response. Once the adaptive immune response starts fighting the dengue infection, the antigens present on virus particles activate B-cells, which mature into plasma cells which then produce antibodies called IgM and IgG [5, 17]. These antibodies travel though the blood stream and bind to the antigens making them noninfectious. The cytotoxic T-cells recognize and kill cells that are infected with pathogens. This is illustrated in Figure 1. The external appearance of this whole process is onset of fever along with symptoms such as headaches, muscle or joint pain, myalgia, arthralgia, and rash which is termed as an acute febrile illness that gets cured within 7–14 days by a complex immune response process [5, 20].

Extensive research on mathematical modeling of dengue epidemiology has been done for the last century [21–26] but only a few models have been developed to study within-host dengue viral dynamics. None of the existing models [5, 6, 18, 27] considered the role that innate immune response plays in clearing the dengue infection until recent published work by [14], introduced innate immune response to a target cell limited model, and showed that only innate immunity is needed to recover the characteristic features of a primary infection.

This study is an attempt to develop a computer simulation model to reproduce the known dynamics of healthy cells, infected cells, virus, B-cells, and immune response. Both innate and humoral immune responses have been incorporated to the model to evaluate the effect of immune response on viral control. This model is merely a conceptual model to capture the qualitative behaviour of virus dynamics. Thus we can then extend this model to fit quantitative data from clinical experiments. In this study, two viral titer peaks were observed during the course of infection. It was found that the innate immune response is responsible for the first rapid viral decline and for the subsequent second peak in viral load. It is also noted that the humoral immune response is needed to eventually clear the virus from the body. Next we identify the significant parameters and carry out a sensitivity analysis to investigate the virus dynamics with respect to parameter variability.

In order to validate the results, we carry out a detailed stability analysis followed by numerical simulations to identify the relevant ranges of key parameters which generates different scenarios of the infection.

Experimental studies have shown that higher viremia titer early in the course of infection is associated with more severe disease [28, 29]. Peak virus titers were 100- to 1000-fold higher for patients who developed severe dengue compared to those with DF [28]. Some studies have shown that antibody dependent enhancement causes severe dengue in secondary infections [5] but [30] found that a significant proportion of primary infections may also cause severe disease indicating a non-ADE mechanism. Also there is evidence that excessive activation of the immune system may lead to a cytokine storm which may cross-react with vascular endothelium and cause increased vascular permeability and vascular leak leading to severe dengue disease [14, 31–33]. Thus by analyzing the sensitivity of the model parameters, we can identify which parameters and parameter values may contribute towards severe dengue.

## 2. Methods

### 2.1. Model Development

Here we develop a model to study the within-host dynamics of dengue infection which incorporates both innate and adaptive immune response. We assume that only one serotype of dengue virus circulates in an infected host and the virus infects monocytes, macrophages, dendritic cells, and hepatocytes in the blood stream. This model is an extension of the model presented in [5] in which only humoral immune response is considered in clearing primary and secondary dengue infection. In acute infection, apart from [14] which describes the role of innate immunity in dengue infection, most influenza models highlight the role of innate immune response on viral control [9, 34, 35].

To model the effects of innate immune response on dengue viral dynamics, we make the assumption that number of activated NK cells is proportional to the level of IFN as in the recently published dengue model [14] and influenza model [9].

### 2.2. The Mathematical Model

The dynamics of healthy cells, infected cells, virus, B-cells, antibodies, and interferon are described in the following system of equations.

**Figure 2:**
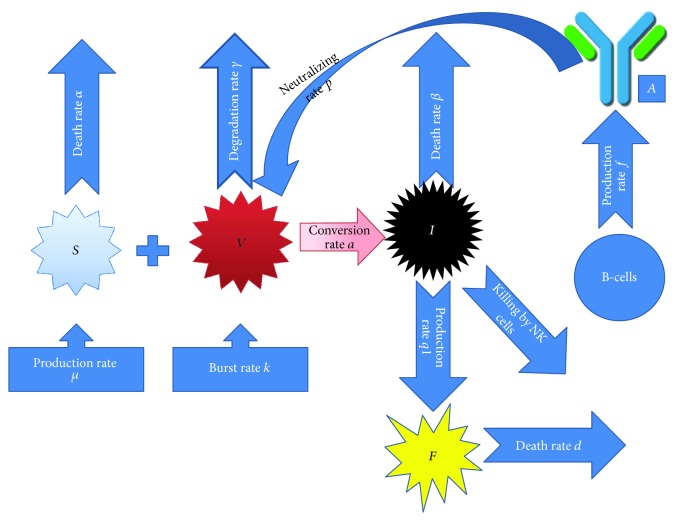
Schematic representation of the mathematical model.

(1) dSdt=μ−αS−aSVdIdt=aSV−βI−ϕIFdVdt=kI−γV−pVAdBdt=η−δB+cBVdAdt=fB−qAV−κAdFdt=q1I−dF,

where *S* indicates susceptible cells (monocytes, macrophages, dendritic cells, hepatocytes, or mast cells), *I* indicates infected cells, *V* indicates dengue virus particles, *B* indicates B-cells, *A* indicates antibodies, and *F* indicates interferon. Susceptible cells are produced at a constant rate *μ* and they die at a rate *α*. Susceptible cells become infected by virus at a rate *aSV*, where *a* is the rate of infection. The infection period is assumed constant given by 1/*β*. The infected cells are killed by NK cells at a rate proportional to *ϕ*. Virus replicates in infected cells and bursts out at a rate *k* and decays at a rate *γ*. Virus is being neutralized by antibodies at a rate *p*. B-cells are produced at a constant rate *η* and die at a rate *δ*. On coming into contact with virus, B-cells become activated at a rate *c*. These activated B-cells then transform into plasma cells (denoted by the same variable as B-cells) which produce antibodies (*A*) at a rate *f*. Antibodies are destroyed by virus at a rate *q* and naturally at a rate *κ*. IFN (*F*) is produced by infected cells at a rate *q*_1_ and decays at a rate *d*. A schematic diagram of model (1) is shown in Figure 2.

### 2.3. Parameters of the Model

Description of model parameters along with baseline parameter values is given in the Nomenclature section. We parametrize the model parameters using a combination of literature estimates [5, 6, 14].

## 3. Results

### 3.1. Numerical Simulation

**Figure 3:**
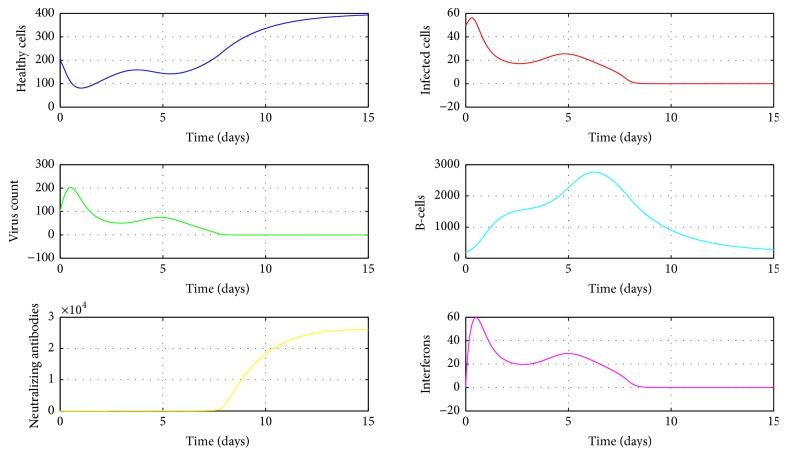
The dynamics of *S*, *I*, *V*, *B*, *A*, *F*. The parameter values used are *μ* = 20, *α* = 0.05, *a* = 0.0013, *β* = 0.5, *κ* = 0.009, *γ* = 0.5, *p* = 0.007, *η* = 10, *δ* = 0.049, *c* = 0.001, *q* = 0.9, *f* = 0.8, *k* = 2, *q*_1_ = 0.8, *d* = 0.7, *ϕ* = 0.002. Initial condition (*S*, *I*, *V*, *B*, *A*, *F*) = (200,50,100,200,0, 0).

A numerical simulation of the model (1) is done in MATLAB using the ODE45 solver and is given in Figure 3. The parameter values used for this simulation are given in the Nomenclature section. In our simulation model, we observe bimodal virus titer peaks. The first peak occurs around day 1 after infection and the second virus peak occurs around day 5. The viral load declines rapidly after the first peak, then generates a second peak around day 5, and finally declines to below detection limit within 7–14 days which is in line with clinical observations [20, 36]. Interferon also displays similar kind of behaviour which is agreeable with experimental studies [30]. However there is a delay in production of antibodies which rises above their limit of detection approximately 4–7 days after infection, consistent with experimental findings [19, 37]. The second viral decline emerges with the generation of antibodies and without antibody mediated immune response the virus will not be cleared from the host.

Next, our aim is to analyze the effects of model parameters on dynamics of dengue infection. For this, we carry out a sensitivity analysis for key parameters such as the infection rate, *a*, antibody production rate, *f*, and virus burst rate, *k*, and consider the mean virus count and the standard deviations that can be used as basis for understanding the patterns of disease severity.

### 3.2. Sensitivity Analysis

A sensitivity analysis of the model was done with respect to *a*, *f*, and *k*. For this, we solve the model for random values of each parameter using explicit Euler method which is described as follows.

Given an initial value problem,

(2) dydt=ft,yt,yt0=y0.

We decide upon what interval we desire to find the solution. Then we subdivide this interval into small lengths of *h*. Then, using the initial condition as our starting point, we generate the rest of the solution by using the iterative formulas:

(3) tn+1=tn+hyn+1=yn+hftn,yn.

The value of *y*_*n*_ is an approximation of the solution to the ODE at time *t*_*n*_. Thus *y*_*n*_ ≈ *y*(*t*_*n*_).

The mean virus count and the standard deviation of the virus count are visualized using MATLAB software package and the algorithm used is given as follows.

*Algorithm for the Sensitivity Analysis*

**Step 1 .**

Input the parameter values except the parameter for which the sensitivity analysis is carried for.

**Step 2 .**

Set up the initial conditions.

**Step 3 .**

Create 1000 random values of the parameter within a specified interval.

 
*p* = (*v* − *u*)*∗*rand(1000,1) + *u*
 
*p* = generates random numbers ∈(*u*, *v*).

**Step 4 .**

Create an iterative loop

 
*N* = number of time intervals
 
*h* = (*t*_max_ − *t*_0_)/*N*
 
**for ***i* = 1 : number of random numbers** do**
 
 **for ***n* = 1 :  *N *** do**
 
    for each *i*
 
    *t*_*n*+1_ = *t*_*n*_ + *h*
 
    *y*_*n*+1_ = *y*_*n*_ + *h∗f*(*t*_*n*_, *y*_*n*_)
 
 **end for**
 
**end for**
  compute mean(*y*)
  compute sd(*y*)

#### 3.2.1. Sensitivity Analysis of Rate of Infection (*a*)

To analyze the sensitivity of the model with respect to the rate of infection (the rate at which healthy cells are converted to infected cells due to interaction with virus particles (*a*)), we solve the model numerically for randomized values of *a*. Using explicit Euler method, we can analyze how sensitive the system reacts to fluctuations in *a*. Here we use initial conditions (*S*, *I*, *V*, *B*, *A*, *F*) = (200,50,100,200,0, 0) and the parameter values used are *μ* = 20, *α* = 0.05, *β* = 0.5, *κ* = 0.009, *γ* = 0.5, *p* = 0.007, *η* = 10, *δ* = 0.049, *c* = 0.001, *q* = 0.9, *f* = 0.8, *k* = 2, *q*_1_ = 0.8, *d* = 0.7, *ϕ* = 0.002.

**Figure 4:**
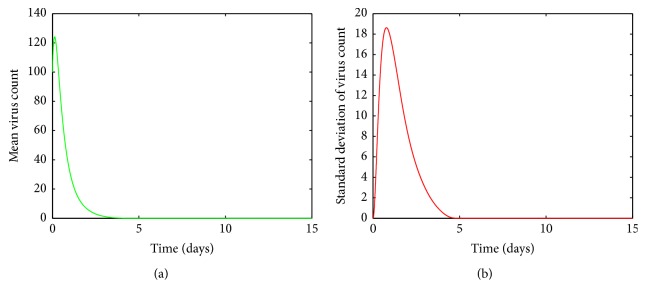
(a) Mean value of the virus count and (b) standard deviation of the virus count for randomized values of *a* between 0.00001 and 0.0005. The parameter values used are *μ* = 20, *α* = 0.05, *β* = 0.5, *κ* = 0.009, *γ* = 0.5, *p* = 0.007, *η* = 10, *δ* = 0.049, *c* = 0.001, *q* = 0.9, *f* = 0.8, *k* = 2, *q*_1_ = 0.8, *d* = 0.7, *ϕ* = 0.002.

**Figure 5:**
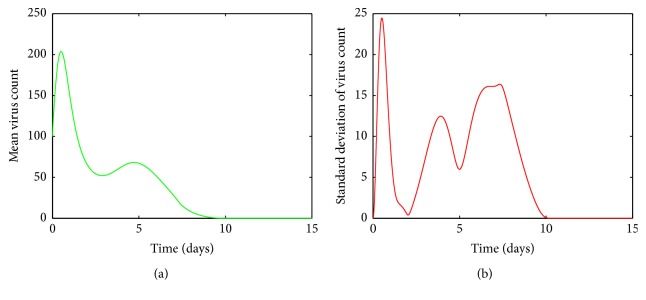
(a) Mean value of the virus count and (b) standard deviation of the virus count for randomized values of *a* between 0.0009 and 0.0018. The parameter values used are *μ* = 20, *α* = 0.05, *β* = 0.5, *κ* = 0.009, *γ* = 0.5, *p* = 0.007, *η* = 10, *δ* = 0.049, *c* = 0.001, *q* = 0.9, *f* = 0.8, *k* = 2, *q*_1_ = 0.8, *d* = 0.7, *ϕ* = 0.002.

**Figure 6:**
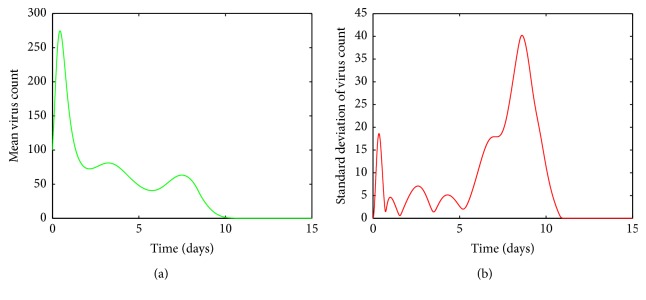
(a) Mean value of the virus count and (b) standard deviation of the virus count for randomized values of *a* between 0.0018 and 0.003. The parameter values used are *μ* = 20, *α* = 0.05, *β* = 0.5, *κ* = 0.009, *γ* = 0.5, *p* = 0.007, *η* = 10, *δ* = 0.049, *c* = 0.001, *q* = 0.9, *f* = 0.8, *k* = 2, *q*_1_ = 0.8, *d* = 0.7, *ϕ* = 0.002.

**Figure 7:**
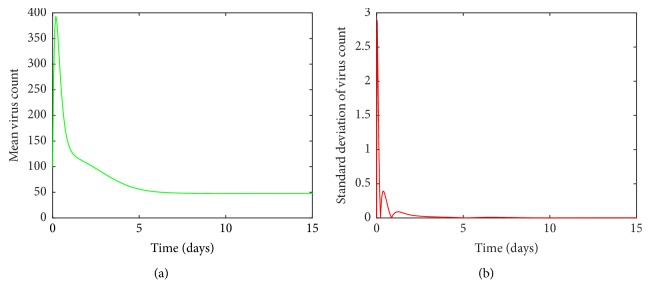
(a) Mean value of the virus count and (b) standard deviation of the virus count for randomized values of *a* between 0.07 and 0.1. The parameter values used are *μ* = 20, *α* = 0.05, *β* = 0.5, *κ* = 0.009, *γ* = 0.5, *p* = 0.007, *η* = 10, *δ* = 0.049, *c* = 0.001, *q* = 0.9, *f* = 0.8, *k* = 2, *q*_1_ = 0.8, *d* = 0.7, *ϕ* = 0.002.

In Figures 4(a), 5(a), 6(a), and 7(a), the mean value of the virus count *V* over 1000 runs of the model (1) is visualized. Figures 4(b), 5(b), 6(b), and 7(b) show the standard deviation of the solutions around the mean value. As seen from Figure 4(a), for small values of *a*, after a first increasing phase, the mean virus count declines rapidly and the infection gets cleared within 15 days. Also it is seen that there are small variations in the virus count, *V*, around the climax (Figure 4(b)). Since there is only one predictable fluctuation and since the standard deviation goes to negligible levels, it is inferred that the system is insensitive to changes in *a* between 00001 and 0.0005.

It is clear from Figure 5(a), for values of *a* between 0.0009 and 0.0018, the viral load increases rapidly and reaches its first peak around day 1. After the peak, the viral load declines and then experiences another peak around day 5. After the second peak, the viral load declines again to its disease free equilibrium.  Figure 5(b) shows there are variations around the means near both peaks. The variations around the two peaks and infection clear-out point are quite high compared to the mean. Thus we can confirm that the system is sensitive for disturbances in *a* in this region. From Figures 6(a) and 6(b), it is seen that for *a* values between 0.0018 and 0.003 there are fluctuations around the mean between the first peak and the third peak. Thus we can infer that the system is sensitive to changes in this region even though the standard deviation of the virus count eventually goes to undetectable levels.

Figure 7(a) shows that, for high values of *a*, the viral load increases rapidly, reaches its peak, and then declines gradually to its endemic equilibrium. Thus the infection does not clear up from the host. Also we can see from Figure 7(b) that the variations in the mean approach zero. Thus we can conclude that the mean virus count converges to some fixed nonzero value.

Further, by comparing Figures 4(a), 5(a), 6(a), and 7(a) it is observable that, for small values of *a*, the infection gets cleared from the host and for larger values of *a*, the infection remains endemic. Thus the model is stable with respect to the infection rate *a*.

#### 3.2.2. Sensitivity Analysis of Production Rate of Antibodies (*f*)

Here the variation of dynamics of (1) with respect to rate of production of antibodies, *f*, is considered. The mean virus count and the standard deviation of the virus count are visualized for different ranges of *f*. The initial values and other parameter values remain the same as in the sensitivity analysis for the infection rate to guarantee comparability.

**Figure 8:**
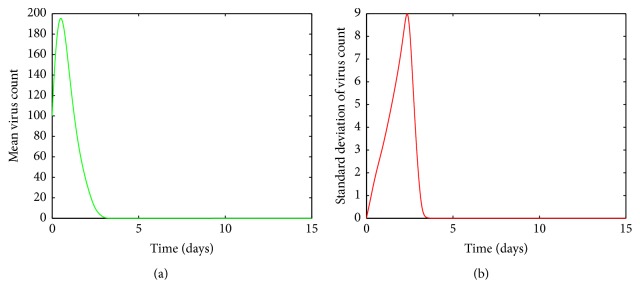
(a) Mean value of the virus count and (b) standard deviation of the virus count for randomized values of *f* between 1.5 and 2.5. The parameter values used are *μ* = 20, *a* = 0.0013, *α* = 0.05, *β* = 0.5, *κ* = 0.009, *γ* = 0.5, *p* = 0.007, *η* = 10, *δ* = 0.049, *c* = 0.001, *q* = 0.9, *k* = 2, *q*_1_ = 0.8, *d* = 0.7, *ϕ* = 0.002.

**Figure 9:**
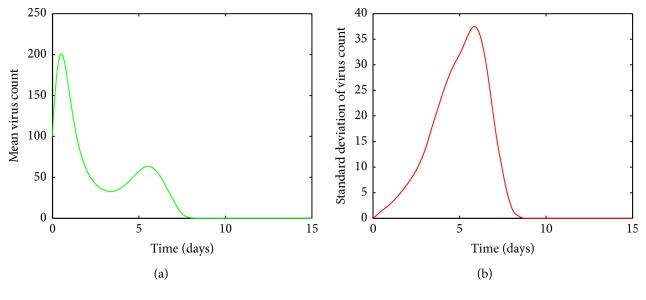
(a) Mean value of the virus count and (b) standard deviation of the virus count for randomized values of *f* between 0.7 and 1.5. The parameter values used are *μ* = 20, *a* = 0.0013, *α* = 0.05, *β* = 0.5, *κ* = 0.009, *γ* = 0.5, *p* = 0.007, *η* = 10, *δ* = 0.049, *c* = 0.001, *q* = 0.9, *k* = 2, *q*_1_ = 0.8, *d* = 0.7, *ϕ* = 0.002.

**Figure 10:**
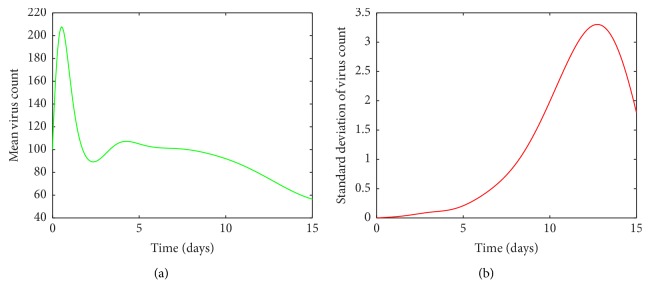
(a) Mean value of the virus count and (b) standard deviation of the virus count for randomized values of *f* between 0.005 and 0.01. The parameter values used are *μ* = 20, *a* = 0.0013, *α* = 0.05, *β* = 0.5, *κ* = 0.009, *γ* = 0.5, *p* = 0.007, *η* = 10, *δ* = 0.049, *c* = 0.001, *q* = 0.9, *k* = 2, *q*_1_ = 0.8, *d* = 0.7, *ϕ* = 0.002.

In Figures 8(a), 9(a), and 10(a), the mean value of *V* over 1000 runs is visualized for different ranges of *f*. As seen in Figure 8(a), for large *f* values, the viral load increases gradually to its peak and then declines rapidly to the disease free equilibrium. It is clearly seen from Figure 8(b) that the standard deviation of the virus count is high around the climax. However, this variation around the mean exists only for a short period and then the standard deviation converges to zero. This confirms that the system is robust towards the variations for high production rate of antibodies.

For *f* between 0.7 and 1.5, it is evident from Figure 9(a) that two peaks occur in the viral load and there is a high standard deviation in the mean virus count near the second peak (Figure 9(b)). These fluctuations remain only for a short period of time and the smooth behaviour of the curve afterward predicts that the system is not very susceptible for disturbances in *f* in this region.

Similarly, it is clear from Figures 10(a) and 10(b) that, for low values of *f*, the infection remains endemic within host and the fluctuations around the mean are very high. From these observations, it can be inferred that the antibody dynamics has a predictable effect on the behaviour of the virus count.

#### 3.2.3. Sensitivity Analysis of Burst Rate of Virus Particles (*k*)

The mean virus count and its standard deviation are depicted for different ranges of virus burst rate, *k*. In each case, the immune response is also drawn for a random value of *k* within each range to observe the immune response to changes in viral load. The initial values remain the same as in previous cases.

**Figure 11:**
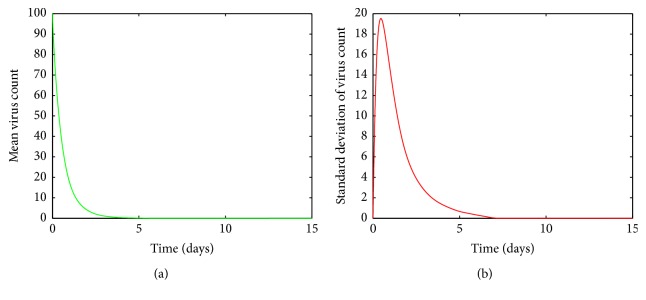
(a) Mean value of the virus count and (b) standard deviation of the virus count for randomized values of *k* between 0.1 and 1. The parameter values used are *μ* = 20, *a* = 0.0013, *α* = 0.05, *β* = 0.5, *κ* = 0.009, *γ* = 0.5, *p* = 0.007, *η* = 10, *δ* = 0.049, *c* = 0.001, *q* = 0.9, *f* = 0.8, *q*_1_ = 0.8, *d* = 0.7, *ϕ* = 0.002.

For small values of *k*, the mean virus count (Figure 11(a)) would yield only one peak and then rapidly converge to the disease free equilibrium. It is clear from Figure 11(b) that there is a high standard deviation around the mean near the climax but the standard deviation rapidly declines to negligible levels after a short time. The immune response to changes in *k*, within this range, is visualized in Figure 15.

**Figure 12:**
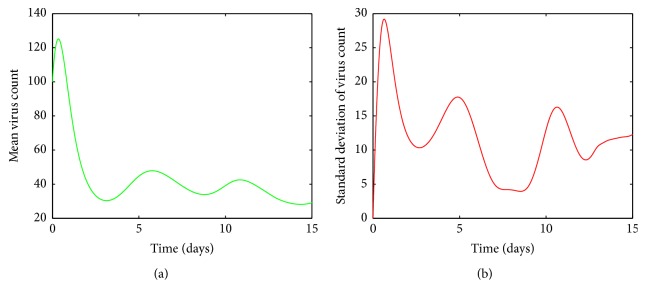
(a) Mean value of the virus count and (b) standard deviation of the virus count for randomized values of *k* between 1 and 1.8. The parameter values used are *μ* = 20, *a* = 0.0013, *α* = 0.05, *β* = 0.5, *κ* = 0.009, *γ* = 0.5, *p* = 0.007, *η* = 10, *δ* = 0.049, *c* = 0.001, *q* = 0.9, *f* = 0.8, *q*_1_ = 0.8, *d* = 0.7, *ϕ* = 0.002.

**Figure 13:**
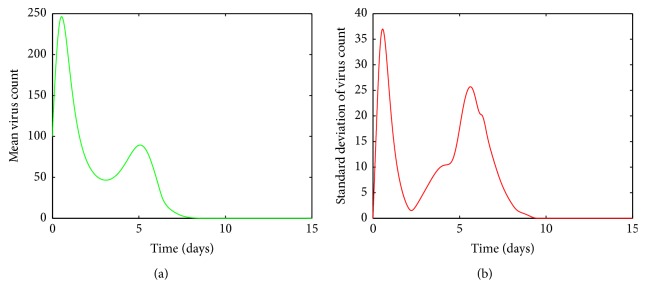
(a) Mean value of the virus count and (b) standard deviation of the virus count for randomized values of *k* between 1.9 and 2.7. The parameter values used are *μ* = 20, *a* = 0.0013, *α* = 0.05, *β* = 0.5, *κ* = 0.009, *γ* = 0.5, *p* = 0.007, *η* = 10, *δ* = 0.049, *c* = 0.001, *q* = 0.9, *f* = 0.8, *q*_1_ = 0.8, *d* = 0.7, *ϕ* = 0.002.

**Figure 14:**
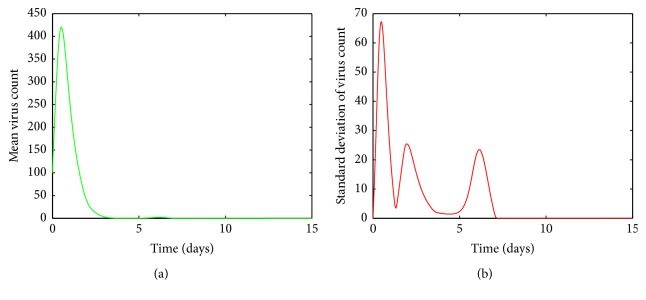
(a) Mean value of the virus count and (b) standard deviation of the virus count for randomized values of *k* between 2.7 and 4. The parameter values used are *μ* = 20, *a* = 0.0013, *α* = 0.05, *β* = 0.5, *κ* = 0.009, *γ* = 0.5, *p* = 0.007, *η* = 10, *δ* = 0.049, *c* = 0.001, *q* = 0.9, *f* = 0.8, *q*_1_ = 0.8, *d* = 0.7, *ϕ* = 0.002.

For *k* between 1 and 1.8, an endemic state in the mean virus count is observed. The standard deviation also varies throughout this period and converges to an endemic equilibrium (Figure 12(b)). Further, from Figure 16, it is observed that both innate immune response and antibody mediated immune response fluctuate within a small range for *k* within this range. For *k* between 1.9 and 2.7, two viral peaks are observed in the mean virus count and if we increase *k* further, only one peak in the mean virus count is seen. This phenomenon is shown in Figures 13(a) and 14(a), respectively. The explanation for this phenomenon could be understood by the immune response visualized in Figures 17 and 18, respectively.

Even though only one peak is observed in the mean virus count for high values of *k*, we see many oscillations in the standard deviation around the mean virus count (Figure 14(b)). Also it can be seen from Figure 13(b) that fluctuations in standard deviation around the mean exist for *k* between 1.9 and 2.7. Thus we can infer that the system is highly sensitive for changes in the parameter *k*.

## 4. Basic Reproduction Number and Stability Analysis

### 4.1. The Basic Reproduction Number

Whether or not the virus can grow and establish a persistent infection depends on the basic reproduction number. The basic reproduction number, *R*_0_, is defined as the average number of infected cells produced by each infected cell when almost all cells are uninfected [38]. The basic reproduction number was calculated using the next generation method and is given by the following equation.

(4) $$\begin{array}{c} R_{0}=\frac{a\delta \kappa \mu k}{\alpha \beta \left(\delta \gamma \kappa +\eta fp\right)}. \end{array}$$

If *R*_0_ < 1, the infection will be cleared ultimately. Initially the virus may spread, but once the immune response becomes fully activated, each infected cell will give rise to less than one infected cell, in which the virus population may decline and die out. If *R*_0_ > 1, the infection will persist and give rise to an endemic equilibrium where the virus count would settle to some nonzero value.

### 4.2. Stability Analysis

In order to discuss the stability of the model, we first solve system (1) for its equilibrium points.

The infection free equilibrium point is obtained when *V*^*∗*^ = 0 and is given by the following equation.

(5) $$\begin{array}{c} E_{1}=\left(\;S^{*},I^{*},V^{*},B^{*},A^{*},F^{*}\;\right)=\left(\;\frac{\mu }{\alpha },0,0,\frac{\eta }{\delta },\frac{f\eta }{\delta \kappa },0\;\right). \end{array}$$

In this case, the virus gets cleared in a short time while the antibodies persist in the body for a long time.

We next study the stability of the infection free steady state. For this, we must first linearize the system about its equilibrium points and the corresponding Jacobian matrix is given by the following equation.

(6) $$\begin{array}{c} J=\left[\begin{array}{cccccc} -\left(\alpha +aV^{*}\right) & 0 & -aS^{*} & 0 & 0 & 0\\ aV^{*} & -\beta -\phi F^{*} & aS^{*} & 0 & 0 & -\phi I^{*}\\ 0 & k & -\left(\gamma +pA^{*}\right) & 0 & -pV^{*} & 0\\ 0 & 0 & cB^{*} & \left(cV^{*}-\delta \right) & 0 & 0\\ 0 & 0 & -qA^{*} & f & -\left(qV^{*}+\kappa \right) & 0\\ 0 & q_{1} & 0 & 0 & 0 & -d \end{array}\right]. \end{array}$$

After substituting the equilibrium points given in (5), the characteristic equation of *J* can be written as in the following equation.

(7) Gλ=d+λκ+λδ+λα+λαδκλ2+αδκβ+γ+αηfpλ+αβδγκ+ηfp−aμkδκ.

The eigenvalues of (7) are

(8) λ1=−dλ2=−κλ3=−δλ4=−αλ5=−b±b2+4α2βδκδγκ+ηfpR0−12αδκwhere  b=αδκβ+γ+αηfp.

It can be seen that all eigenvalues will be negative if *R*_0_ < 1. Thus the infection free equilibrium is stable if *R*_0_ < 1.

The basic reproduction number was calculated for different rates of infection and is depicted in Figures 19(a) and 19(b). As seen from Figures 4(a), 5(a), and 6(a), the mean virus count converges to 0 for *a* between 0.00001 and 0.003 and in this case it is seen by Figure 19(a) that *R*_0_ < 1.

We can prove that model (1) has a unique endemic equilibrium if *R*_0_ > 1. The analytical proof is not shown here. In Figure 19(b),  *R*_0_ was computed for randomized values of *a* within the range 0.07–0.1 and it was observed that *R*_0_ > 1. Also it can be seen by Figure 20 for *a* between 0.07 and 0.1, susceptible cells, *S*, infected cells, *I*, dengue virus particles, *V*, B-cells, *B*, antibodies, *A*, and interferon, *F*, converge to their endemic equilibrium values marked with an asterisk. Thus numerically it can be seen that the endemic equilibrium is stable for *R*_0_ > 1 and the virus count settles to some nonzero value.

Similarly for *f* between 0.7 and 2.5, we can see from Figure 21(a) that *R*_0_ < 1 and for *f* between 0.005 and 0.01, *R*_0_ > 1(Figure 21(b)). From Figure 22, it is clear that the endemic equilibrium is stable for *R*_0_ > 1 and the virus count converges to some nonzero value.

## 5. Discussion

In this study, a conceptual simulation model was developed using a system of ordinary differential equations to understand the dynamics of dengue virus. The ordinary differential equations describe the dynamical behaviour of healthy cells, infected cells, virus count, B-cells, antibodies, and interferon. We were able to generate bimodal virus titer peaks by adding the effect of IFN. A simple immune response model cannot produce a rapid viral decline after the first peak unless the death rate of infected cells is chosen to be very large [9]. But this will completely eliminate the second peak which is observed in this study. Thus the first viral decline in this model can be explained by the killing of infected cells by IFN activated NK cells during the innate immune response. The proposed simulation model has made the assumption that level of activated NK cells is proportional to the level of IFN.

As the innate immune response weakens, the killing of infected cells by NK cells lapses, which would increase the level of infected cells and give rise to a second viral titer peak. After reaching the second peak around day 5 after infection, the virus count declined to below detection level within 7–14 days which is agreeable with clinical observations [20, 36]. The second viral decline is mainly due to the antibody mediated immune response which is needed in the model for the eventual viral clearance. Numerical simulation presented in Figure 3 confirmed this phenomenon. Also it can be seen from Figure 3 that a positive correlation exists between virus count and IFN which is in line with experimental studies [30].

We then examined the sensitivity of the viral load with respect to several key parameters including *a*, *f*, and *k* that are thought to play a significant role in dengue infection. According to clinical expertise, a person with dengue infection is advised to bed rest and frequent review of fluid management is done. One can argue that this can affect model parameters such as the infection rate or the virus burst rate of a host. For example, it might be the case that, by bed rest, the infection rate or the virus burst rate can be lowered. On the other hand, if we consider a parameter such as production rate of healthy cells, *μ*, it might depend on age and sex. So if we arbitrarily change it without considering different groups, it might not display correct results. Thus the proposed qualitative model can be enhanced to fit with clinical data if the significant parameters are identified.

For very low values of the infection rate, *a*, the second viral peak is not visible because of cytolysis of infected cells by NK cells. Thus the infection is controlled by innate immune response. However antibody mediated immune response is needed for the ultimate virus clearance. For moderate infection rates, both innate immune response and antibody mediated immune response are needed to clear the virus from the host. As we increase *a*, the number of peaks in viral load also increases. For higher values of *a*, both immune responses are unable to clear the infection from the host and the infection remains endemic. In order to validate the results, the basic reproduction number, *R*_0_, was computed for different ranges of *a* and it was observed that, for small values of *a*, *R*_0_ < 1, where the infection would eventually die out and for high values of *a*, *R*_0_ > 1. It is clear from Figure 20, for high *a* values (*R*_0_ > 1), the virus count converges to some nonzero value.

By looking at Figures 5(b) and 6(b) it can be seen that the standard deviation of the mean virus count fluctuates within the first ten days and then converges to zero. But this does not mean that patients with such infection rates can be neglected, as we have to greatly care for them within the first ten days as their conditions can worsen. However from Figure 4(b) it can be concluded that we do not have to worry about patients with small infection rates because even though the standard deviation of the virus count is relatively high near the climax, the smooth behaviour of the curve and decreasing rapidly to zero makes it negligible. Thus it can be concluded that the system is highly sensitive to changes in *a*.

The same pattern of behaviour was observed with respect to antibody production rate, *f*. Smaller production rates generated an endemic equilibrium in the virus count and high fluctuations in the standard deviation. Thus both innate and antibody immune responses were unable to curb the infection. In this case *R*_0_ > 1 and from Figure 22 it is clear that the endemic equilibrium is stable for small *f* values. For moderate and high values of *f*, the mean virus count displayed two peaks and one peak, respectively, and the standard deviations around the means displayed a predictable pattern and converged to zero ultimately. Higher production rate of antibodies eliminated the second peak as antibody mediated immune response alone controlled the infection. For these two cases, *R*_0_ was computed and was less than one (Figure 21(a)) leading to an infection free steady state. Thus it can be concluded that the production rate of antibodies has a significant effect on the model but displays a predictable behaviour.

**Figure 15:**
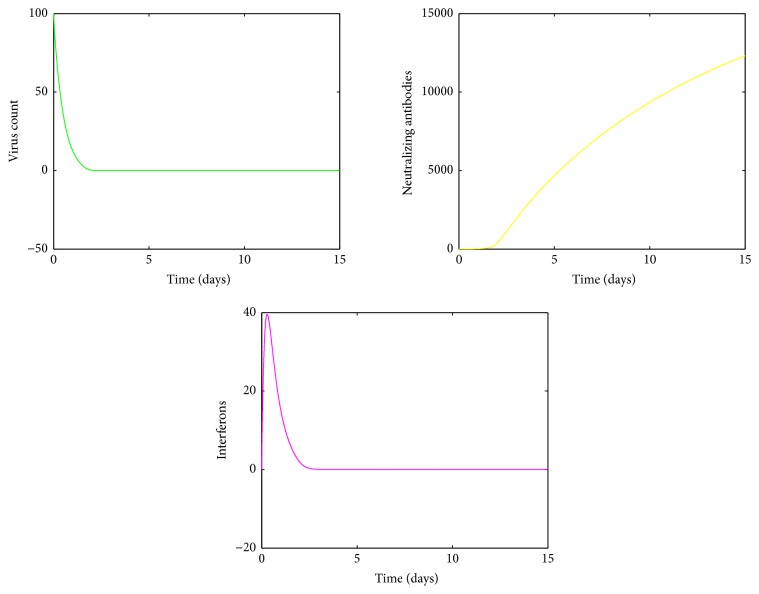
Immune response for *k* = 0.5.

**Figure 16:**
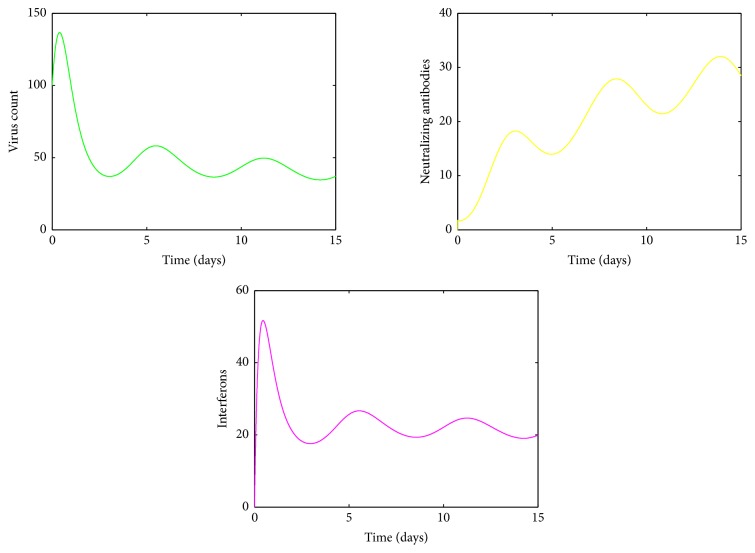
Immune response for *k* = 1.5.

**Figure 17:**
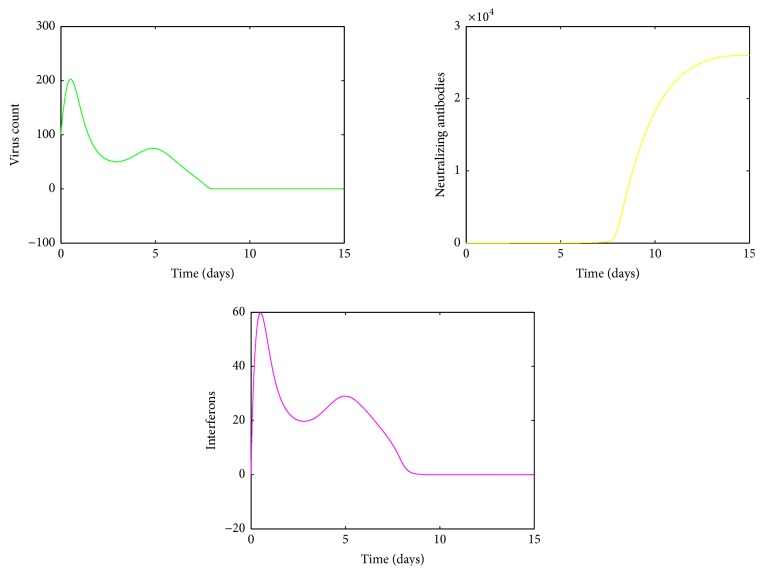
Immune response for *k* = 2.

**Figure 18:**
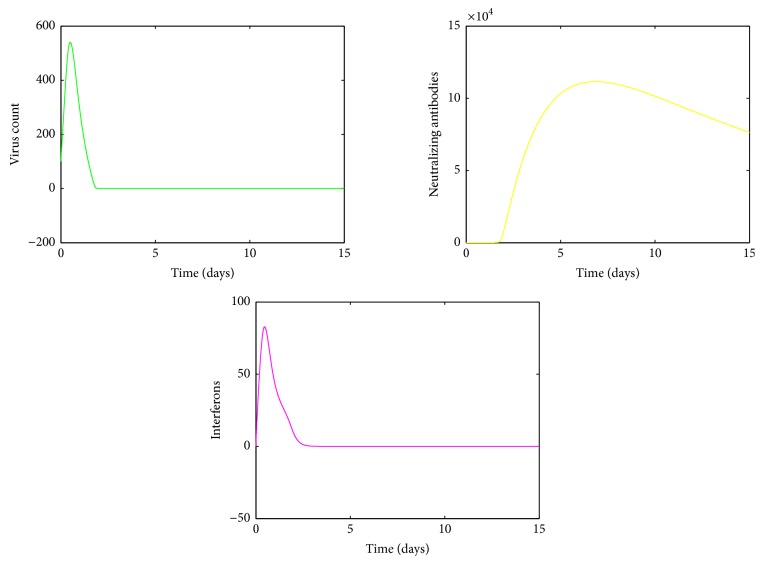
Immune response for *k* = 4.

**Figure 19:**
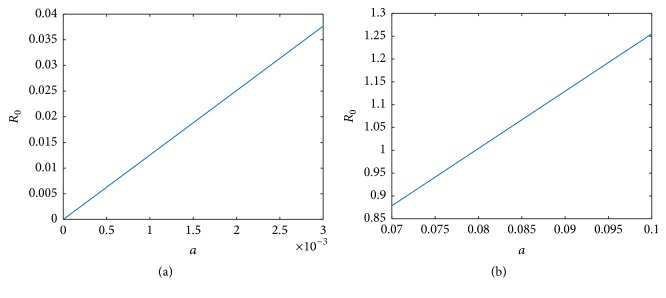
(a) *R*_0_ for randomized values of *a* between 0.00001 and 0.003 and (b) *R*_0_ for randomized values of *a* between 0.07 and 0.1. The parameter values used are *μ* = 20, *α* = 0.05, *β* = 0.5, *κ* = 0.009, *γ* = 0.5, *p* = 0.007, *η* = 10, *δ* = 0.049, *c* = 0.001, *q* = 0.9, *f* = 0.8, *q*_1_ = 0.8, *d* = 0.7, *ϕ* = 0.002.

**Figure 20:**
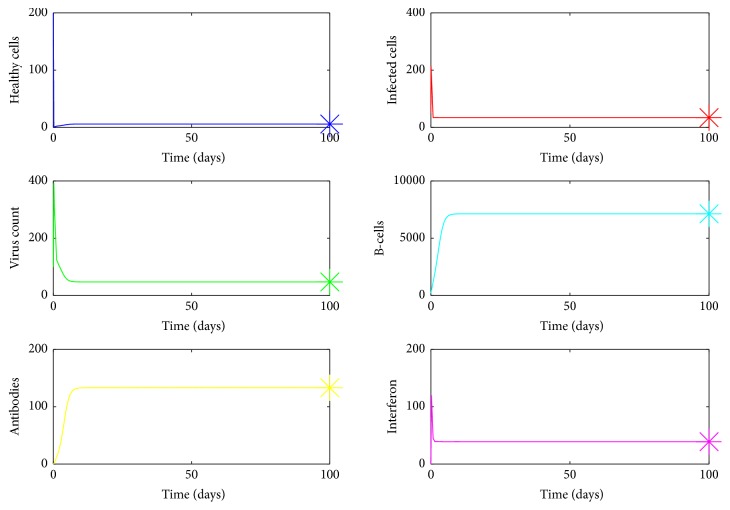
Endemic equilibrium. In this case, *R*_0_ > 1. The equilibrium points are marked with an “*∗*”. The parameter values used are *μ* = 20, *a* = 0.07, *α* = 0.05, *β* = 0.5, *κ* = 0.009, *γ* = 0.5, *p* = 0.007, *η* = 10, *δ* = 0.049, *c* = 0.001, *q* = 0.9, *f* = 0.8, *q*_1_ = 0.8, *d* = 0.7, *ϕ* = 0.002.

**Figure 21:**
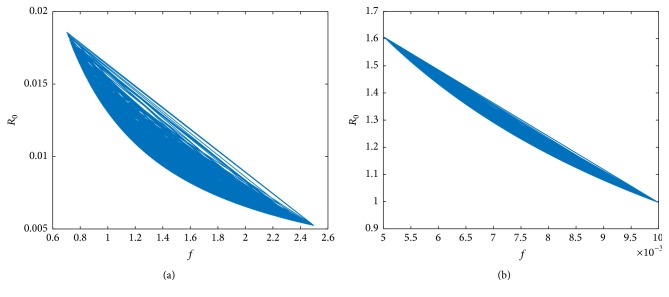
(a) *R*_0_ for randomized values of *f* between 0.7 and 2.5 and (b) *R*_0_ for randomized values of *f* between 0.005 and 0.01. The parameter values used are *μ* = 20, *a* = 0.0013, *α* = 0.05, *β* = 0.5, *κ* = 0.009, *γ* = 0.5, *p* = 0.007, *η* = 10, *δ* = 0.049, *c* = 0.001, *q* = 0.9, *q*_1_ = 0.8, *d* = 0.7, *ϕ* = 0.002.

**Figure 22:**
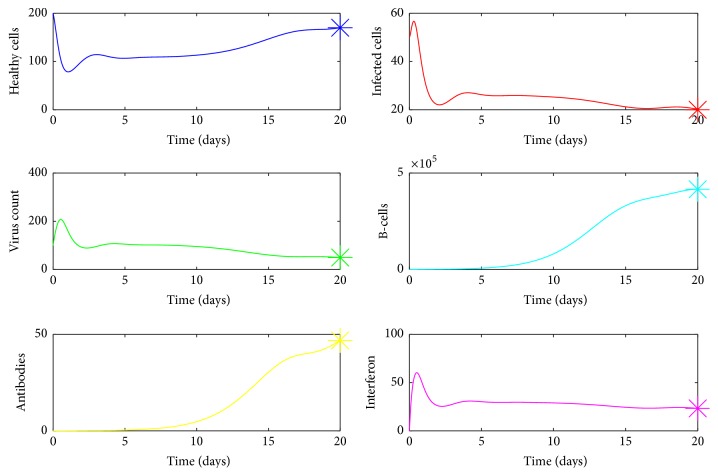
Endemic equilibrium. In this case, *R*_0_ > 1. The equilibrium points are marked with an “*∗*”. The parameter values used are *μ* = 20, *a* = 0.0013, *α* = 0.05, *β* = 0.5, *κ* = 0.009, *γ* = 0.5, *p* = 0.007, *η* = 10, *δ* = 0.049, *c* = 0.001, *q* = 0.9, *f* = 0.005, *q*_1_ = 0.8, *d* = 0.7, *ϕ* = 0.002.

A different sensitivity pattern was observed for changes in virus burst rate, *k*. The human immune system is an enormously complex interrelated system. When the virus burst rate *k* is small, there is only a less virus count. Since B-cells are activated by virus, the number of B-cells is proportional to the virus count. Therefore for low *k* values, there is only a fewer number of B-cells, thus less antibodies. Hence the first peak is controlled by the innate immune system and the antibodies would eventually lead to virus clearance, whereas when *k* is large, there is a higher viral count and proportionally a higher antibody count. The first peak is controlled by innate immunity and by antibody mediated immune response and this high antibody count eliminates the second viral peak. This phenomenon can be seen in Figures 15 and 18, respectively. For *k* values between 1 and 1.8, an oscillatory behaviour in the virus count is observed. The reason can be explained by Figure 16. When the viral load decreases, IFN would also decrease, since there is a positive correlation between IFN and virus count [30]. When the number of virus counts is small, the antibody level in human body would also be small. Hence both innate and antibody immune response are unable to control the virus count, making it rise up. Then IFN would also proportionally increase and decrease the virus count, thus making less number of antibodies as the virus count is proportional to number of antibodies. Thus both IFN and antibodies would fluctuate within a small range but are unable to control the virus.

When *k* is large enough, from Figure 18 a high viremia titer peak and excessive activation of the immune response can be seen. According to [28, 29], high viremia peaks in the early course of infection are associated with severe disease. Also in [14] it is noted that excessive activation of the immune response may lead to severe disease. Thus it can be inferred that when the virus burst rate is high, a person can experience severe dengue. Oscillations in the standard deviation of the virus count during the first few days as shown in Figure 14(b) make it a worrisome condition even though the standard deviation goes to zero eventually. Thus with close monitoring and extra care, the spread of the virus can be controlled and eliminated from the host.

## 6. Conclusions

In this study, a computer simulation model based on an ODE dynamical system was developed to understand the dynamics of dengue virus. A sensitivity analysis of some key parameters was carried out to better understand the development of severe disease and what role model parameters play in severe dengue disease. Also the stability of the infection free steady state and endemic steady state of the model were discussed with respect to the basic reproduction number, *R*_0_. Then a detailed numerical analysis was done to identify the relevant parameter values that leads to infection free or infectious states.

Two viral titer peaks were observed in our simulation model and was able to show that cytolysis of infected cells by NK cells during innate immune response is responsible for the rapid viral decline after the first peak and for the subsequent second peak. Humoral immune response is needed for the second viral decline and to eventually clear the virus.

Sensitivity analysis of the parameters *a*, *f*, and *k* was carried out to predict the viral load changes and to analyze the dynamics of immune responses. As the infection rate *a* is increased, the number of peaks in the viral load would increase leading to a virus persistence state. In this case, it was observed that the basic reproduction number *R*_0_ > 1. Oscillations in the standard deviations of the mean virus count for all different values of *a* implied that the model is highly sensitive to changes in the parameter *a*.

As the antibody production rate *f* increased, the number of virus titer peaks and the mean viral load decreased. This implies that the antibody dynamics has a significant effect on the viral load. Also for high *f* values, *R*_0_ < 1 and the virus count converged to zero. The fluctuations in standard deviation around the mean, for different values of *f*, displayed a predictable behaviour.

As the virus burst rate is increased, the number of virus titer peaks decreased. For high *k* values, only one peak was observed in the viral load. As the burst rate is high, the virus count is high and since activated B-cells are proportional to the virus count, there is a high B-cell count and proportionally a high antibody count. Thus the viral load is controlled by IFN and antibodies and because of high immune response, the second peak is entirely eliminated. However since this situation displays a high initial viral peak and excess activation of immune response, it can represent a severe dengue condition. For intermediate *k* values, we observed oscillatory behaviour in the virus count, IFN, and antibody count. Figure 16 represents this situation which leads to a virus persistence state. Thus it cannot be predicted that as *k* increases, the virus count would lead to an endemic state, which is a false statement. This explains the fact that human body is an interrelated system and increasing one parameter may not lead to an adverse effect.

## Nomenclature

*μ*: Production rate of healthy cells, 20 cells/(day·*μ*l)
*α*: Death rate of healthy cells, 0.05/day
*a*: Rate at which healthy cells are converted to infected cells due to their interaction with virus particles, 0.0013 cells/(day·*μ*l)
*β*: Death rate of infected cells, 0.5/day
*ϕ*: Rate at which infected cells are being killed by NK cells, 0.002/day
*k*: Burst rate of virus particles, 2/day
*γ*: Rate at which virus particles degrade, 0.5/day
*p*: Rate at which virus particles are neutralized by antibodies, 0.007 cells/day
*η*: Production rate of B-lymphocytes, 10 cells/(day·*μ*l)
*δ*: Death rate of B-lymphocytes, 0.049/day
*c*: Rate at which B-lymphocytes are stimulated by virus particles, 0.001 cells/day
*f*: Rate at which stimulated B-cells (Plasma cells) produce antibodies, 0.8/day
*q*: Rate at which antibody virus complex affects the antibody growth, 0.9/day
*κ*: Rate at which free antibodies degrade, 0.009/day
*q*_1_: Production rate of IFN, 0.8 cells/(day·*μ*l)
*d*: Rate of decay of IFN, 0.7/day.
