# Supplementary material for: Simulation Model for Dynamics of Dengue with Innate and Humoral Immune Responses
Source: Comput Math Methods Med. 2018 Apr 11;2018:8798057. doi: 10.1155/2018/8798057 (PMC5925133; doi:10.1155/2018/8798057)
Supplement: Supplementary Materials — MATLAB codes for the sensitivity analysis are provided in the supplementary materials. [file 8798057.f1.docx]

**Additional File 1 (MATLAB codes)**

**Sensitivity**

Finding the mean and standard deviation for different a values.

%rand is an obsolete random number generator that generates uniformly distributed outputs in the open interval (0, 1).

rand('state',2) % initializes the random number generator to the Nth integer state.

tmax=15; % Final Time in days

N=100000; % Number of time intervals

dt=tmax/N; % step size in time

t(1)=0;

%% Parameters

mu=20;

alpha=0.05;

beta=0.5;

kappa=0.009;

gamma=0.5;

p=0.007;

eta=10;

delta=0.049;

c=0.001;

q=0.9;

f=0.8;

k=2;

q1=0.8;

theta=0.7;

phi=0.002;

%% Arrays to hold the solutions

S=zeros(300,N+1); % healthy cells

I=zeros(300,N+1); % Infected cells

V=zeros(300,N+1); % Virus

B=zeros(300,N+1); % B cells

Z=zeros(300,N+1); % Antibodies

F=zeros(300,N+1); % Interferon

Ma=zeros(N+1,6); % Matrix to hold the solutions

%% Initial Conditions

S(:,1)=200;

I(:,1)=50;

V(:,1)=100;

B(:,1)=200;

Z(:,1)=0;

F(:,1)=0;

u=0.003; % choose different values

v=0.01; % choose different values

a=(v-u).*rand(300,1) + u; % generates random numbers between u and v.

b=a';

%% Loop for Euler method

for i=1:300

for n=1:N

t(n+1)=t(n)+dt;

S(i,n+1)=S(i,n)+(mu-b(i)*S(i,n)*V(i,n)-alpha*S(i,n))*(100/11)*dt; I(i,n+1)=I(i,n)+(b(i)*S(i,n)*V(i,n)-beta*I(i,n)-phi*I(i,n)*F(i,n))*(100/11)*dt;

V(i,n+1)=V(i,n)+(k*I(i,n)-gamma*V(i,n)-p*V(i,n)*Z(i,n))*(100/11)*dt; B(i,n+1)=B(i,n)+(eta-delta*B(i,n)+c*B(i,n)*V(i,n))*(100/11)*dt;

Z(i,n+1)=Z(i,n)+f*B(i,n)-q*Z(i,n)*V(i,n)-kappa*Z(i,n))*(100/11)*dt;

F(i,n+1)=F(i,n)+(q1*I(i,n)-theta*F(i,n))*(100/11)*dt;

end

end

m=mean(V);

s=std(V);

%% plotting mean and standard deviation

plot(t,m,'g','LineWidth',5);

xlabel('time(days)'),ylabel('mean virus count')

hold on

figure()

plot(t,s,'r','LineWidth',5);

xlabel('time(days)'),ylabel('standard deviation of virus count')
